# Establishment of a 6-signature risk model associated with cellular senescence for predicting the prognosis of breast cancer

**DOI:** 10.1097/MD.0000000000035923

**Published:** 2023-11-17

**Authors:** Xiu-Xia Zhang, Xin Yu, Li Zhu, Jun-Hua Luo

**Affiliations:** a Department of Thyroid and Breast Surgery, Linping Campus, The Second Affiliated Hospital of Zhejiang University School of Medicine, Hangzhou, Zhejiang, China; b Pathology Department, Linping Campus, The Second Affiliated Hospital of Zhejiang University School of Medicine, Hangzhou, Zhejiang, China.

**Keywords:** biomarkers, breast cancer, prognosis, risk model, senescence

## Abstract

This study focused on screening novel markers associated with cellular senescence for predicting the prognosis of breast cancer. The RNA-seq expression profile of BRCA and clinical data were obtained from TCGA. The pam algorithm was used to cluster patients based on senescence-related genes. The weighted gene co-expression network analysis was used to identify co-expressed genes, and LASSO-Cox analysis was performed to build a risk prognosis model. The performance of the model was also evaluated. We additionally explored the role of senescence in cancer development and possible regulatory mechanism. The patients were clustered into 2 subtypes. A total of 5259 genes significantly related to senescence were identified by weighted gene co-expression network analysis. LASSO-Cox finally established a 6-signature risk model (ADAMTS8, DCAF12L2, PCDHA10, PGK1, SLC16A2, and TMEM233) that exhibited favorable and stable performance in our training, validation, and whole BRCA datasets. Furthermore, the superiority of our model was also observed after comparing it to other published models. The 6-signature was proved to be an independent risk factor for prognosis. In addition, mechanism prediction implied the activation of glycometabolism processes such as glycolysis and TCA cycle under the condition of senescence. Glycometabolism pathways were further found to negatively correlate with the infiltration level of CD8 T-cells and natural killer cells but positively correlate with M2 macrophage infiltration and expressions of tissue degeneration biomarkers, which suggested the deficit immune surveillance and risk of tumor migration. The constructed 6-gene model based on cellular senescence could be an effective indicator for predicting the prognosis of BRCA.

## 1. Introduction

Breast cancer (BRCA) has become common cancer that affects women’s health all around the world. BRCA is mainly categorized into 3 subtypes, namely positive hormone receptor (HR) such as estrogen receptor (ER^+^) or progesterone receptor (PR^+^), positive human epidermal receptor 2 (HER2^+^), and triple-negative breast cancer (TNBC) (ER^−^, PR^−^, HER2^−^).^[1]^ The most prevalent subtype is the BRCA expressing HR which accounts for 60% to 70% of cases with BRCA in developed countries exclusively in premenopausal women. TNBCs, accounting for approximately 15% to 20% of cases, typically behave more aggressively than other subtypes of BRCA and have worse overall survival (OS).^[2]^ The therapeutic strategy for patients of BRCA varies in different molecular characteristics, and TNBC treatment is the most challenging compared to other subgroups of BRCA.

BRCA is a multi-factorial disease, and there is no clear clarification regarding its etiology. BRCA presents low immunogenicity due to low mutation rates and reduced lymphocyte infiltration.^[3]^ In addition, the importance of cellular senescence in cancer development has also been paid more and more attention. Cellular senescence is a stable cell cycle arrest that limits the proliferative life span.^[4]^ Several apparent characteristics appear in senescent cells, including elevated expression of the cell cycle inhibitor and secretion of cytokines, metalloproteinases (MMPs), and senescence-associated secretory phenotype.^[5]^ Because of its antiproliferative ability, senescence is also regarded as a potent antitumor mechanism.^[4]^ In addition, senescence also contributes to the immunesurveillance of cancer cells. For example, senescent hepatocytes can promote the infiltration of CD4-positive T cells.^[6]^ The ligands of natural killer (NK) cell receptors were upregulated in senescent cells, increasing the NK-cell-mediated cytotoxicity.^[7]^ The senescent cells also expressed the chemokine (C-C motif) ligand 2 (CCL2), contributing to the recruitment of NK cells.^[8]^ It follows that senescence elicits the infiltration of innate immune cells, which mediates the direct tumoricidal effects.^[9]^

However, cellular senescence may also modulate tumorigenesis. Several chemotherapeutic drugs can induce senescence of human cells, but senescent cells can chronically promote local and systemic inflammation that causes or exacerbates many side effects of the chemotherapy.^[10]^ In addition, the senescent cells can secret the MMPs, IL-6, and IL-1β, which facilitates the tumor growth and development.^[10]^ In terms of breast cancer (BRCA), prolonged exposure of BRCA cells to IL-6 induces the appearance of new senescent cells, which favors the acquisition of EMT and stem-like features, thus increasing tumor aggressiveness.^[11]^ Breast cancer cells exposed to the senescence-associated secretory phenotype also strongly upregulates the lipocalin-2, which was critical for the increased migration in BRCA cells.^[12]^ Regarding the tumor microenvironment, the secreted IL-6 during senescence also recruits the myeloid suppressive cells to inhibit T-cells function.^[13]^ It follows that senescence plays an important role as a double-edged sword in cancer development. Identifying significant senescence-related biomarkers and exploring their potential for cancer development are of great significance. To date, the contribution of cellular senescence to BRCA remains elusive.

Machine-learning-derived signatures are useful in predicting cancer prognosis and guiding the cancer treatment. This study established and validated a risk model based on cellular senescence-related genes and evaluated its prognostic value in BRCA. Then, the potential mechanism associated with senescence biomarkers in BRCA was explored. We expected our findings to provide a new perspective for predicting prognosis and mechanism revelation in BRCA.

## 2. Methods

### 2.1. The obtaining of senescence-related genes

In this study, we detected 12 senescence-related pathways from Molecular Signature Database (MSigDB, http://www.gsea-msigdb.org/gsea/msigdb/index.jsp) and PathCards database (https://pathcards.genecards.org/). The 12 pathways included 679 genes. After removing the duplicated genes, 381 senescence-related genes were finally identified. Related pathways and corresponding gene numbers were presented in Table 1.

**Table 1:** The senescence-related pathways.

Pathway name	Gene number
S1. Stress_induced_premature_senescence	8
S2. Cellular Senescence	198
S3. DNA Damage Telomere Stress Induced Senescence	80
S4. Oncogene Induced Senescence	35
S5. Oxidative Stress Induced Senescence	126
S6. Glycolysis in senescence	11
S7. Kynurenine pathway and links to cell senescence	23
S8. NAD metabolism in oncogene-induced senescence and mitochondrial dysfunction-associated senescence	23
S9. Prostaglandin and leukotriene metabolism in senescence	31
S10. Sphingolipid metabolism in senescence	28
S11. TCA cycle in senescence	10
S12. Senescence and autophagy in cancer	105
Total	679
After removing duplicated genes	381

### 2.2. Cluster analysis and molecular subtypes identification

First, we obtained the gene expression profile and clinical data of patients with BRCA from The Cancer Genome Atlas (TCGA) database (https://portal.gdc.cancer.gov/). The clinical data included the patient’s age, T/N/M stage, clinical stage, OS time, and OS status. We removed samples without clinical follow-up information and genes with an expression value of 0 in more than half of the samples. We also excluded the male patients in this study. Then, we extracted the expression profile of 381 genes related to senescence from the TCGA-BRCA dataset. A univariate Cox regression analysis on all BRCA patients was performed to explore the correlation of 381 genes with OS, and significant genes with *P* < .05 were subsequently used to cluster the patients by Consensus Cluster Plus. The parameters for cluster analysis included: calculation for clustering distance (Pearson), maximum size (10), frequency in sampling (10), sampling proportion (0.8), and clustering method (pam). The indicators for cluster analysis contained the relative change in area under the cumulative distribution function curve, sample clustering consistency, and consensus clustering matrix. In addition, we performed the Kaplan–Meier (KM) analysis to explore the survival difference between clusters.

### 2.3. WGCNA and significant modules determination

The weighted gene co-expression network analysis (WGCNA) based on the whole samples was then performed to screen the coexpressed coding genes and modules. We calculated the median absolute deviation of each gene, excluded the top 50% of the minimum median absolute deviation genes, and eliminated the outlier genes and samples using the goodSamplesGenes method of the R software package WGCNA. Further, WGCNA was used to construct a scale-free co-expression network. The β is a soft-thresholding parameter that could emphasize strong correlations between genes and penalize weak correlations. The filtering principle of soft threshold was to make the constructed network more consistent with the characteristics of a scale-free network. The weighted adjacency matrix was transformed into a topological overlap matrix to estimate its connectivity in the network. To classify genes with similar expression profiles into gene modules, average linkage hierarchical clustering was conducted according to the topological overlap matrix -based dissimilarity measure with a minimum size (gene group) of 60 for the genes dendrogram, and the sensitivity was set as 3. We also merged modules with distances of <0.25. Finally, the module genes with significant correlation with phenotypes were selected for further analysis.

### 2.4. LASSO analysis on module genes

After the WGCNA analysis, we conducted the Least absolute shrinkage and selection operator (LASSO) analysis to filter the most important biomarkers. Before the LASSO analysis, the patients with BRCA were randomly divided into a training set and validation set in a 6:4 ratio. A total of 1077 samples with BRCA were included, and 646 patients were assigned to the training set and 431 patients were assigned into the validation set. There were no differences in terms of age, gender, and clinical stage between training and validation sets. Further, we performed the following analyses in training set. We first performed a univariable Cox regression analysis to explore the correlation of important module genes with OS. Then the genes with *P* < .01 were enrolled into the LASSO analysis to narrow down the candidate genes. LASSO is a linear regression method using L1-regularization and it generates a penalty function, aiming to compress the redundant variable coefficients into 0 and identify the valuable variables. The LASSO analysis can solve the problems of severe collinearity and overfitting.

### 2.5. Establishment of risk model associated with senescence for predicting the OS

After LASSO, several candidate genes were selected. Then an initial risk model was constructed in a training set according to the gene expression and coefficients within the optimal LASSO model. Then the receiver operating characteristic (ROC) and KM analyses were performed to assess the performance of the model. Further, we performed the multivariable Cox regression analysis to filter the target genes among LASSO analysis. According to the Cox coefficients and expression value of independent prognostic factors, we established a final risk model and calculated the risk score. The correlation between risk score and OS was explored by KM analysis. The performance of the risk score for predicting the survival of patients was compared among training set, validation set, and whole TCGA-BRCA dataset. In addition, this study also compared the performance of our risk model with other models in published articles by ROC analysis, KM curve, C-index value, and decision curve analysis (DCA).

### 2.6. Clinical value evaluation of risk model

The independent prognostic value of the final risk model in whole patients with BRCA was assessed by multivariable Cox regression analysis after integrating the patient’s age, T/N/M/ stage, and clinical stage. The nomogram model was then established based on the results of Cox regression. The ROC and KM analyses were used to assess the performance of the nomogram model. Further, we performed a subgroup analysis to evaluate the correlation between risk model and survival by stratifying the patients with age (age ≤ 55 vs age > 55) and clinical stage (I + II vs III + IV). In addition, we also compared the risk score difference among different age, T stage, N stage, M stage, and clinical stage groups.

### 2.7. Potential mechanism associated with the risk model

By the whole BRCA expresssion profile, we first performed the single sample Gene Set Enrichment Analysis (ssGSEA) and obtained the activity score of 12 senescence-related pathways, then revealed the correlation of risk score with pathway activity score. We also revealed the activity difference of 12 pathways between high and low-risk score groups. Further, we obtained the score of 187 KEGG pathways in BRCA by ssGSEA and assessed the correlation of risk score with KEGG pathways. In addition, we explored the potential of senescence on cancer progression from aspects of immune evasion, tissue degeneration, and chronic inflammation. The immune score and ssGSEA were all based on the expression profile of BRCA.

### 2.8. Clinical value analysis of each signature in the risk model

The KM survival analysis was used to explore the correlation of each signature with the OS time of patients with BRCA. The mRNA expression of each signature in tumor and normal tissues were detected. Their protein expression was also evaluated in the Human Protein Atlas database (https://www.proteinatlas.org/).

### 2.9. Statistical analysis

All data were analyzed with SPSS and R packages. Student *t* test and one-way ANOVA were used to compare continuous data. The chi-square test and Fisher test were used to compare qualitative data. The survival difference between groups was compared with the log-rank test. The Cox regression analysis was used to analyze the correlation of variables with OS. The Pearson method was used to explore the correlation between continuous variables. The area under the curve was used as the indicator of the ROC curve. Nomogram analysis was used to evaluate the prediction performance of the model on the survival probability. The CIBERSORT algorithm was used to obtain the infiltration level of 24 immune cells. The *P* < .05 was considered statistically significant.

## 3. Results

### 3.1. Cluster analysis and identification of molecular subtypes

**Figure 1.**
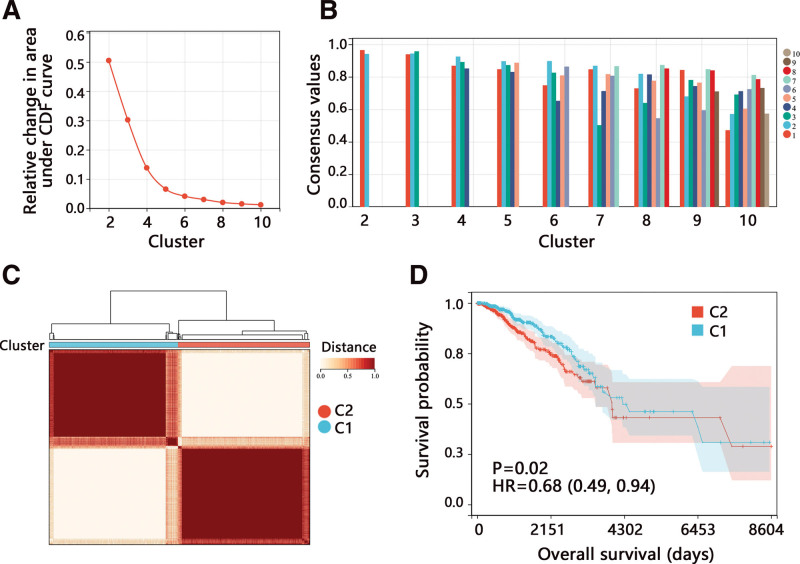
Cluster analysis in BRCA patients with 41 prognosis and senescence-related genes. (A) Relative change in area under the cumulative distribution function (CDF) curve for k = 2–10. (B) Sample clustering consistency. (C) Consensus clustering matrix for k = 2. (D) Survival difference between 2 clusters. HR, hazard ratio.

Among 381 senescence-related genes, 4 genes were not found in the TCGA-BRCA dataset. A total of 377 senescence-related genes were included in this study. Through univariate Cox regression analysis, 41 genes with *P* < .05 were found to be related to the OS (data not shown). Then the patients were clustered with 41 genes. According to the results of cluster analyses, all the BRCA patients were finally clustered into 2 subtypes (Fig. 1A–C). The KM analysis showed the survival difference between the 2 clusters, and the C1 subgroup had a better prognosis than C2 (Fig. 1D, *P* = .02).

### 3.2. WGCNA and identification of significant modules

**Figure 2.**
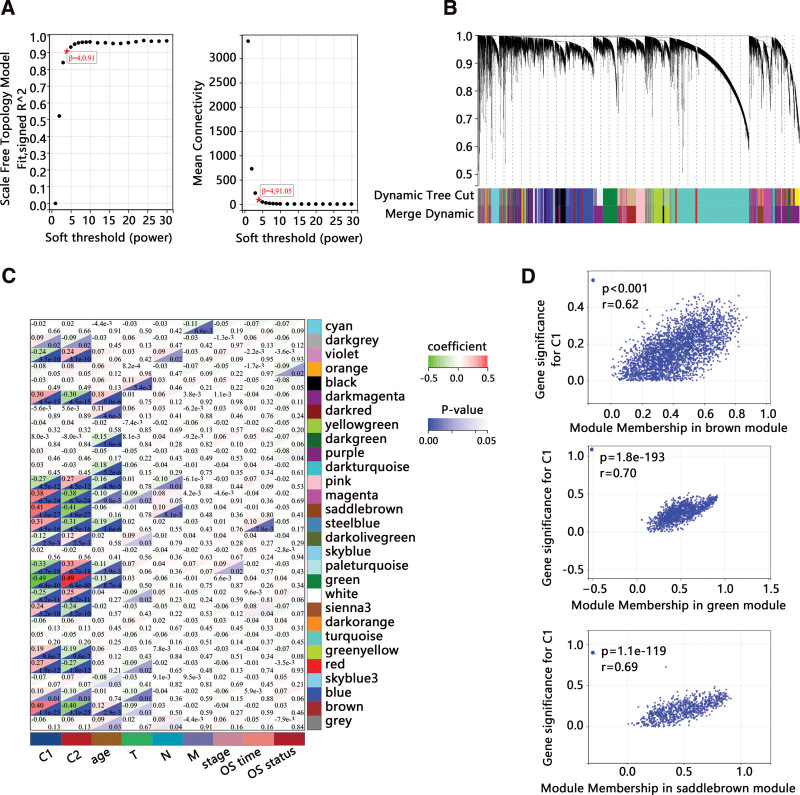
The WGCNA network and identification of significant modules in BRCA. (A) Cluster analysis and soft-thresholding powers. (B) 29 co-expressed modules. (C) Correlations of 29 modules with clinical variables and clusters. (D) Correlation of gene significance (GS) with module membership (MM). BRCA = breast cancer.

The WGCNA was then performed on 57,015 genes in the whole BRCA dataset. Removing the genes with an expression value of 0 in more than half of the samples, 28,508 genes were retained. Then the samples were clustered using hierarchical clustering. To ensure that the network was scale-free, β was set at 4 (Fig. 2A). We next calculated the dissimilarity of module eigengenes, chose a cut line for the module dendrogram and merged some modules. Finally, 29 coexpressed modules were obtained (Fig. 2B). Correlations of 29 modules with age, clinical stage, N stage, T stage, M stage, C1, and C2 were further analyzed (Fig. 2C), in which modules significantly correlated with C1 and C2 were brown (3113 genes), green (1302 genes), and saddle brown (844 genes) modules. The 3 modules contained 5259 genes. The correlations of gene significance with 3 module memberships were presented in Figure 2D.

### 3.3. LASSO-Cox regression analysis on module genes and risk model establishment

We then filtered the significant prognostic genes among 5259 genes. Before analysis, we randomly divided all the patients into validation and training sets in a 6:4 ratio. The distribution of BRCA patients in validation and training sets was presented in Table 2. All of the clinical characteristics showed no differences between validation and training sets.

**Table 2:** The distribution of BRCA patients in validation and training sets.

Variables	Subgroups	Training (N = 646)	Validation (N = 431)	*P*
Age (years)	≤55	289	174	.156
	>55	357	257	
T stage	1	156	122	.464
	2	383	238	
	3	83	54	
	4	22	16	
N stage	0	302	207	.126
	1	212	142	
	2	69	50	
	3	49	26	
M stage	0	531	359	.126
	1	16	5	
Clinical stage	I	106	75	.384
	II	367	244	
	III	144	99	
	IV	15	4	
OS status	0	551	378	.261
	1	95	53	
OS time (days)		1236.12 ± 47.66	1233.30 ± 56.35	.970

**Figure 3.**
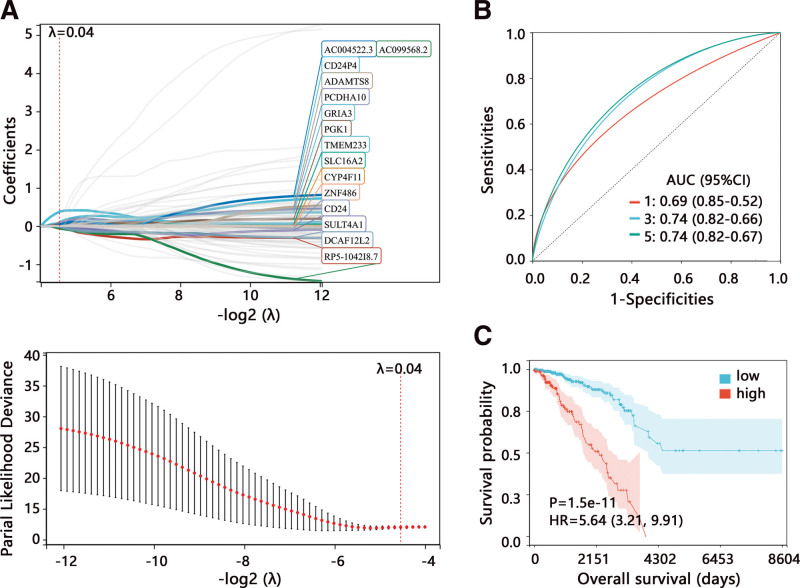
The identification of prognosis-related genes in the training set. (A) LASSO model establishment. (B) ROC analysis and (C) Kaplan–Meier survival analysis on risk score of 15-signature. AUC = area under curve; CI = confidence interval; HR = hazard ratio; LASSO = least absolute shrinkage and selection operator; ROC = receiver operating characteristic.

Then, we firstly used the Cox regression analysis to filter the prognosis-related genes among 5259 genes in the training set. After that, 89 genes with *P* < .01 were detected and enrolled in further LASSO analysis. We integrated the survival data, survival status, and expression value of 89 genes, and performed the LASSO regression analysis. We set the 10-fold cross-validation to obtain the optimal LASSO model. When the lambda (λ) value was 0.043, 15 target markers were included in the final LASSO model (Fig. 3A). A initial risk model of 15-signature was then constructed and risk score were calculated according to the LASSO coefficients and gene expression value. The ROC analysis showed the prediction performance of 15-signature on 1, 3, and 5-year survival of patients (Fig. 3B). The KM analysis indicated that the high-risk group had shorter OS time compared with the low-risk group (Fig. 3C).

Subsequently, we performed the multivariable Cox regression analysis on 15 markers in training set to filter the independent prognostic factor. However, 3 markers were lnRNAs among 15 markers. We excluded the 3 lnRNAs and only performed the multivariable Cox regression analysis on 12 mRNA again. Through multivariable Cox regression with the Forward-Wald method, 6 independent prognostic factors were identified, and 6-gene risk model was established. We also used the other 6 methods to filter the variables that enter the equation (such as Forward-Conditional, Backward-LR), and constructed the corresponding risk model. Further, we performed the ROC analysis to compare the performance of these different models (these data not shown). The results indicated that the risk model under the Forward-Wald method had the best prediction performance. Therefore, we finally established the 6-gene risk model from Forward-Wald Cox regression. The Forward-Wald Cox regression filtered 6 independent factors (Table 3), namely ADAMTS8, DCAF12L2, PCDHA10, PGK1, SLC16A2, and TMEM233.

**Table 3:** The multivariable Cox regression analysis on OS.

Factors	β	HR (95%CI)	*P*
ADAMTS8	0.166	1.181 (1.108, 1.258)	<.001
DCAF12L2	−1.027	0.358 (0.168, 0.762)	.008
PCDHA10	0.802	2.231 (1.489, 3.341)	<.001
PGK1	0.590	1.804 (1.279, 2.543)	.001
SLC16A2	0.390	1.477 (1.179, 1.849)	.001
TMEM233	0.717	2.047 (1.336, 3.138)	.001

ADAMTS8 = ADAM metallopeptidase with thrombospondin type 1 motif 8, CI = confidence interval, DCAF12L2 = DDB1 and CUL4 associated factor 12 like 2, HR = hazard ratio, PCDHA10 = protocadherin alpha 10, PGK1 = phosphoglycerate kinase 1, SLC16A2 = solute carrier family 16 member 2, TMEM233 = transmembrane protein 233.

**Figure 4.**
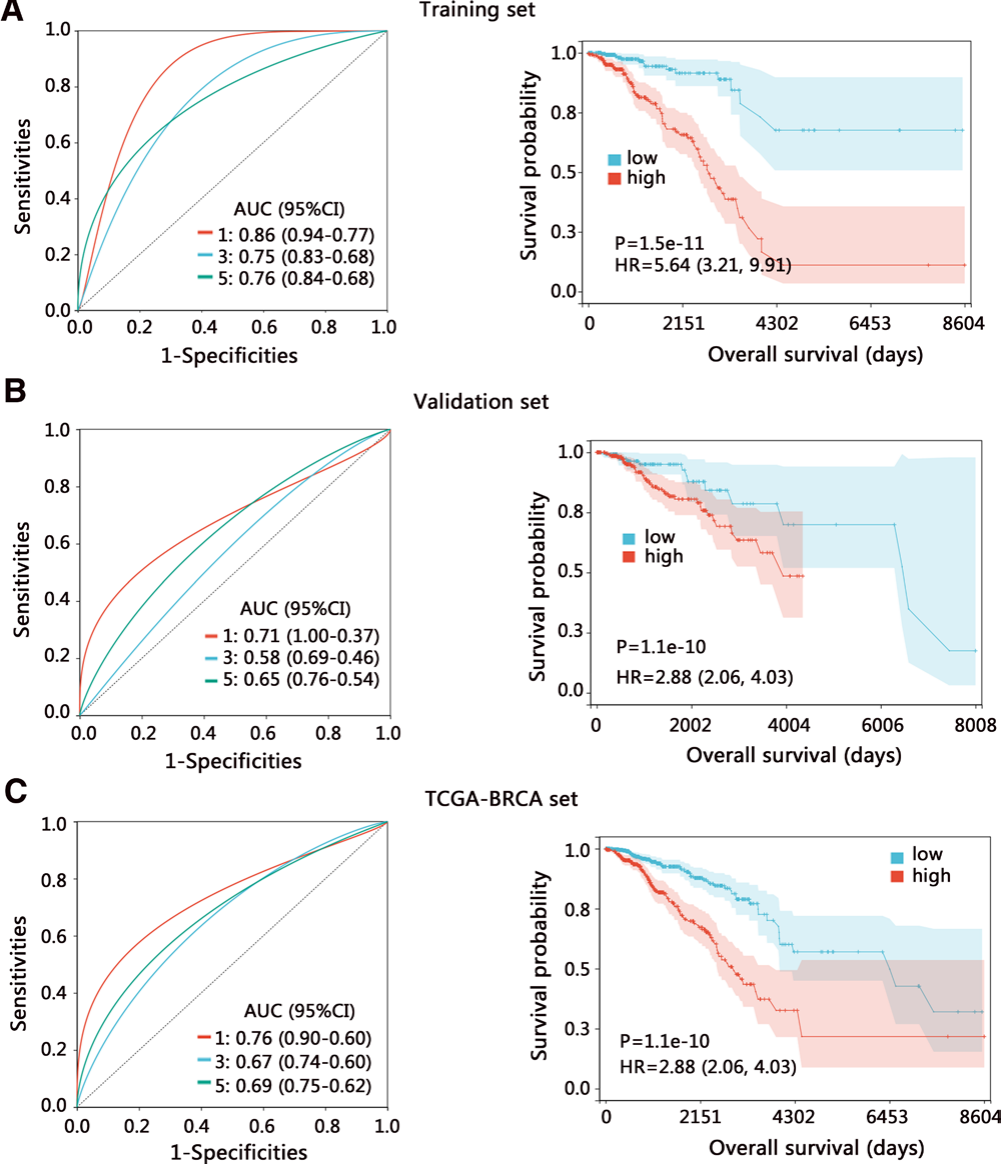
The performance assessment of the 6-signature risk model. ROC and Kaplan–Meier survival analyses on risk model in (A) training set, (B) validation set, and (C) whole TCGA-BRCA set. AUC = area under curve; BRCA = breast cancer; CI = confidence interval; HR = hazard ratio; ROC = receiver operating characteristic; TCGA = The Cancer Genome Atlas.

The formula of the risk model of 6-signature was as follows: risk score = 0.166*ADAMTS8−1.027*DCAF12L2 + 0.802*PCDHA10 + 0.590*PGK1 + 0.390*SLC16A2 + 0.717*TMEM233. Through ROC analysis, we found that the prediction performance in training set of 6-signature was superior to that of initial 15-signature. Therefore, 6-signature model can be regarded as the optimal risk model. The ROC analysis showed that the 6-signature risk model had favorable prediction performance on the 1, 3, and 5-year survival in the training set, and the area under the curve was 0.86, 0.75, and 0.76, respectively (Fig. 4A). The survival analysis showed that patients with high-risk score had a poorer prognosis (HR = 5.64, *P* < .001). We also performed the ROC and Kaplan–Meier survival analyses in validation and whole TCGA-BRCA datasets and evaluated the performance of the risk model (Fig. 4B and C). Our results indicated the favorable performance of the 6-signature risk model to distinguish the BRCA patients.

**Figure 5.**
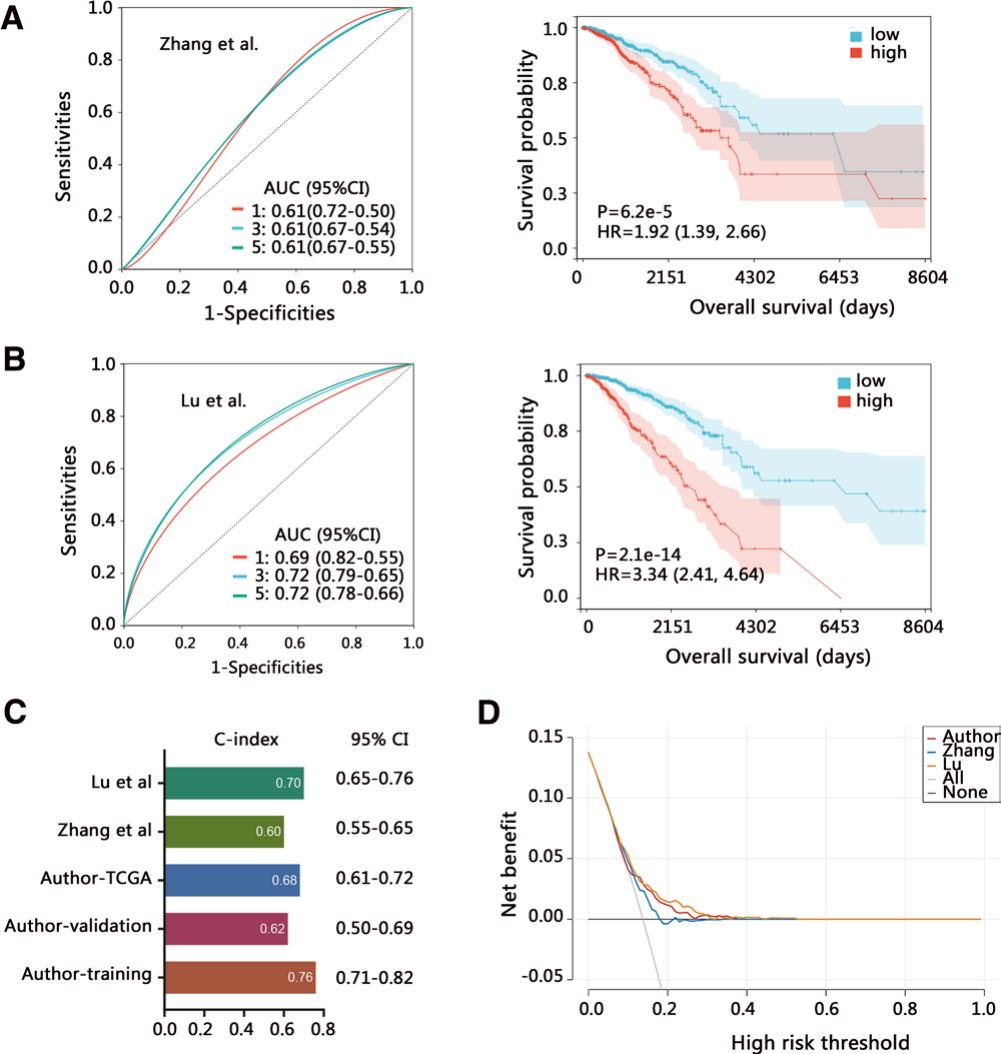
Comparison of our risk model with other signatures. The ROC and Kaplan–Meier analyses on the established risk model in the published (A) Zhang et al and (B) Lu et al studies. (C) The C-index value and (D) DCA curve of different signatures. The author’s risk model in D referred to the whole TCGA-BRCA dataset. AUC = area under curve; BRCA = breast cancer; CI = confidence interval; DCA = decision curve analysis; HR = hazard ratio; ROC = receiver operating characteristic; TCGA = The Cancer Genome Atlas.

We also compared the prediction performance of our risk model on OS with other published signatures. Zhang et al established a 3-signature model associated with pyrimidine metabolism, and Lu et al developed a ferroptosis-related 9-gene model. The ROC analysis showed that our risk model had better performance than the Zhang et al model for predicting 1, 3, and 5-year survival (Fig. 5A), and it was also superior to Lu et al model for predicting the 1-year survival (Fig. 5B). Zhang et al (HR = 1.92, *P* < .001) and Lu et al (HR = 3.34, *P* < .001) studies also reported the negative correlation of risk score with OS of patients. Further, we found that the C-index value of the risk model in our training set was the highest, and it was similar between our whole TCGA-BRCA dataset and the Lu et al study (Fig. 5C). We also conducted the DCA on the risk model and compared the clinical net benefit, finding that our model and Lu et al model achieved more net benefit than Zhang et al risk model (Fig. 5D). These results highlighted the better performance of our risk model.

### 3.4. Clinical value evaluation of the 6-signature risk model in BRCA

This study further explored the prognostic value of the 6-signature risk model in the whole TCGA-BRCA dataset after considering the clinical characteristics by Cox regression analyses. The univariable Cox regression analysis showed that all the variables were significantly associated with the OS (Table 4, all *P* < .001). The risk score was still independently associated with survival in the multivariable Cox regression analysis (HR = 1.857, *P* < .001).

**Table 4:** The Cox regression analysis on OS in the TCGA-BRCA dataset.

	Univariable		Multivariable	
	HR (95%CI)	*P*	HR (95% CI)	*P*
Age	1.036 (1.022, 1.050)	<.001	1.033 (1.018, 1.047)	<.001
T stage	1.473 (1.204, 1.801)	<.001		
N stage	1.623 (1.367, 1.927)	<.001		
M stage	4.589 (2.706, 7.814)	<.001		
Clinical stage	2.164 (1.723, 2.718)	<.001	1.995 (1.597, 2.491)	<.001
6-gene risk score	2.078 (1.689, 2.556)	<.001	1.847 (1.475, 2.314)	<.001

**Figure 6.**
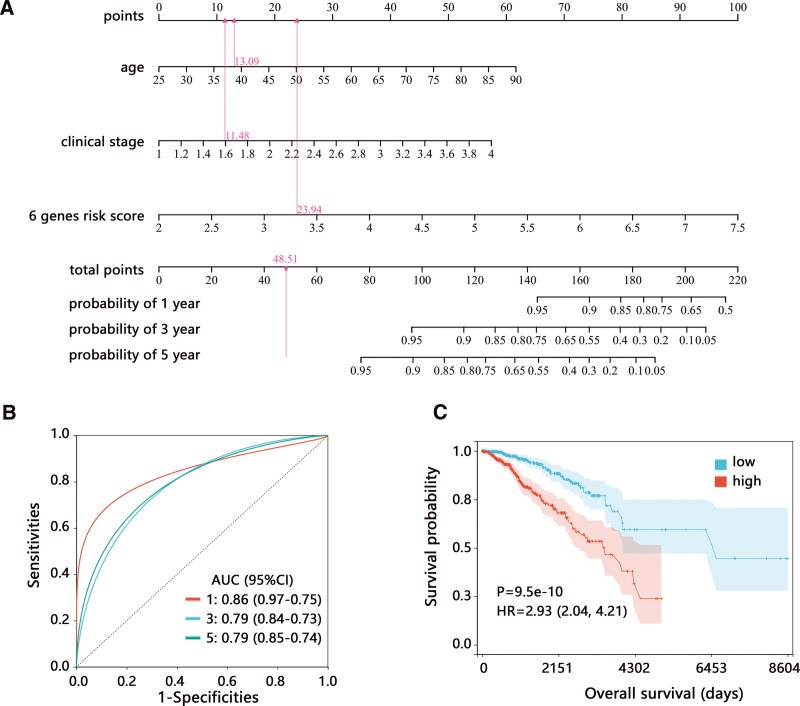
Establishment of comprehensive nomogram model in the whole TCGA-BRCA dataset. (A) Nomogram model for predicting the 1, 3, and 5-year survival probabilities. (B) ROC analysis on nomogram model. (C) Kaplan–Meier analysis on the score of the nomogram model. AUC = area under curve; BRCA = breast cancer; CI = confidence interval; HR = hazard ratio; ROC = receiver operating characteristic; TCGA = The Cancer Genome Atlas.

Based on the multivariable Cox regression analysis, we established a comprehensive nomogram model containing independent prognostic factors of age, clinical stage, and risk score. The comprehensive nomogram model was found to predict a 0.05 to 0.95 survival probability of patients (Fig. 6A), and the 6-signature risk model made the largest contribution. The ROC analysis showed the favorable performance of the comprehensive model in predicting survival (Fig. 6B). Kaplan–Meier analysis also indicated the negative correlation of nomogram score with OS of patients (Fig. 6C).

**Figure 7.**
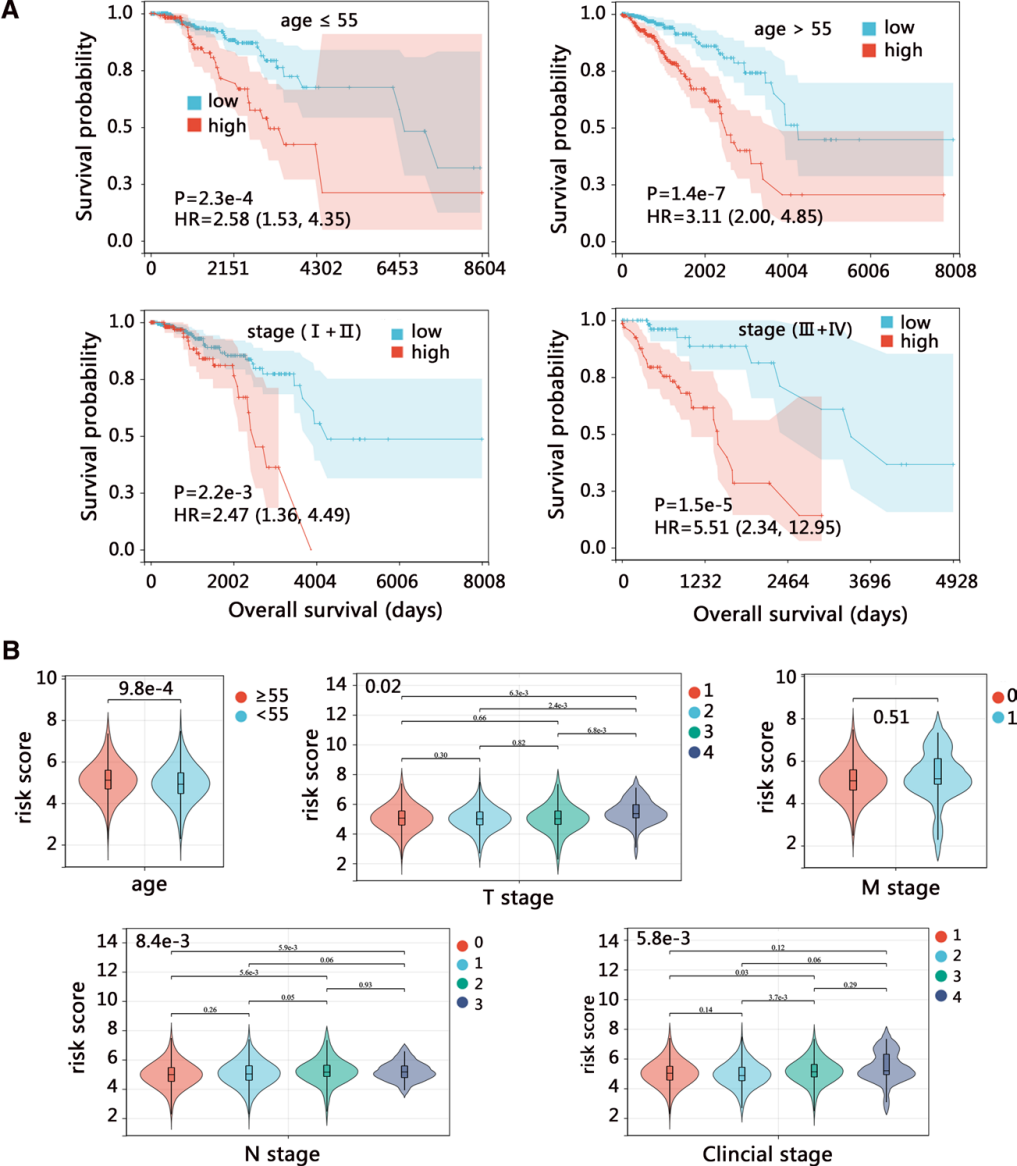
The subgroup analyses on 6-signature risk score in the whole TCGA-BRCA dataset. (A) The Kaplan–Meier survival analysis in BRCA patients stratified by age and clinical stage. (B) The difference in risk score regarding different clinical characteristics. BRCA = breast cancer; TCGA = The Cancer Genome Atlas.

Due to the important role of the 6-signature risk model in BRCA, we detailly explored its function in subgroup populations. The Kaplan–Meier analysis showed a significant negative correlation of risk score with the OS of patients in all subgroups (Fig. 7A, all *P* < .01). Difference comparison indicated that a higher risk score was observed in patients with age ≥ 55 years, T4, N3, and clinical stage IV (Fig. 7B). It followed that risk score might be related to the BRCA progression. We found no difference in risk score between M0 and M1 groups.

### 3.5. The potential mechanism associated with the 6-signature risk model in BRCA

**Figure 8.**
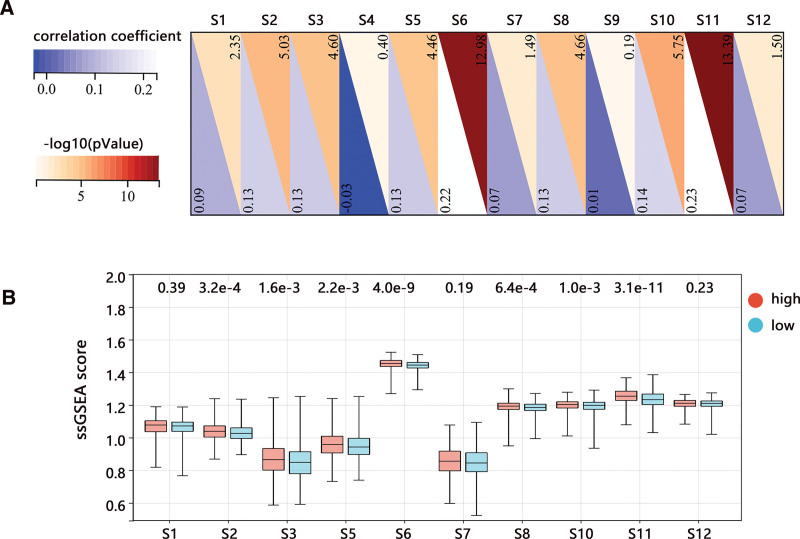
Correlation analysis between risk score and 12 senescence-related pathways in BRCA. (A) Pearson correlation analysis. (B) The difference in ssGSEA score of pathways between low and high-risk score groups. BRCA = breast cancer; ssGSEA = single sample Gene Set Enrichment Analysis.

We then explored the detailed relationship of risk score with the 12 senescence-related pathways. Pearson analysis showed that 10 pathways were significantly related to the risk score (Fig. 8A). The pathways of S6 (Glycolysis in senescence) and S11 (TCA cycle in senescence) showed a larger positive correlation with the risk score. In addition, the ssGSEA score of S6 and S11 pathways were higher than that of other pathways (Fig. 8B). These results implied the correlation of senescence with glycometabolism in BRCA.

Further, we explored the potential KEGG pathways associated with a risk score. A total of 187 KEGG pathways were detected and the top 18 terms were presented in Table 5. KEGG analysis showed that risk score was positively related to the 18 pathways, and the majority of pathways referred to the metabolism, especially the glycometabolism. According to senescence and KEGG pathways, we speculated that the cellular senescence was increased in the high-risk group and the process of glycometabolism was enhanced, which increased the risk of cancer progression. The poorer prognosis of patients with high-risk scores has supported our speculation indeed.

**Table 5:** KEGG pathways associated with the risk score.

N	Pathway name	N	Pathway name
1	N_Glycan_Biosynthesis	10	Drug_Metabolism_Other_Enzymes
2	Fructose_And_Mannose_Metabolism	11	Steroid_Hormone_Biosynthesis
3	Terpenoid_Backbone_Biosynthesis	12	O_Glycan_Biosynthesis
4	Steroid_Biosynthesis	13	Protein_Export
5	Pentose_And_Glucuronate_Interconversions	14	Ubiquitin_Mediated_Proteolysis
6	Starch_And_Sucrose_Metabolism	15	Abc_Transporters
7	Vibrio_Cholerae_Infection	16	Retinol_Metabolism
8	Porphyrin_And_Chlorophyll_Metabolism	17	TCA_Cycle
9	Ascorbate_And_Aldarate_Metabolism	18	Riboflavin_Metabolism

**Figure 9.**
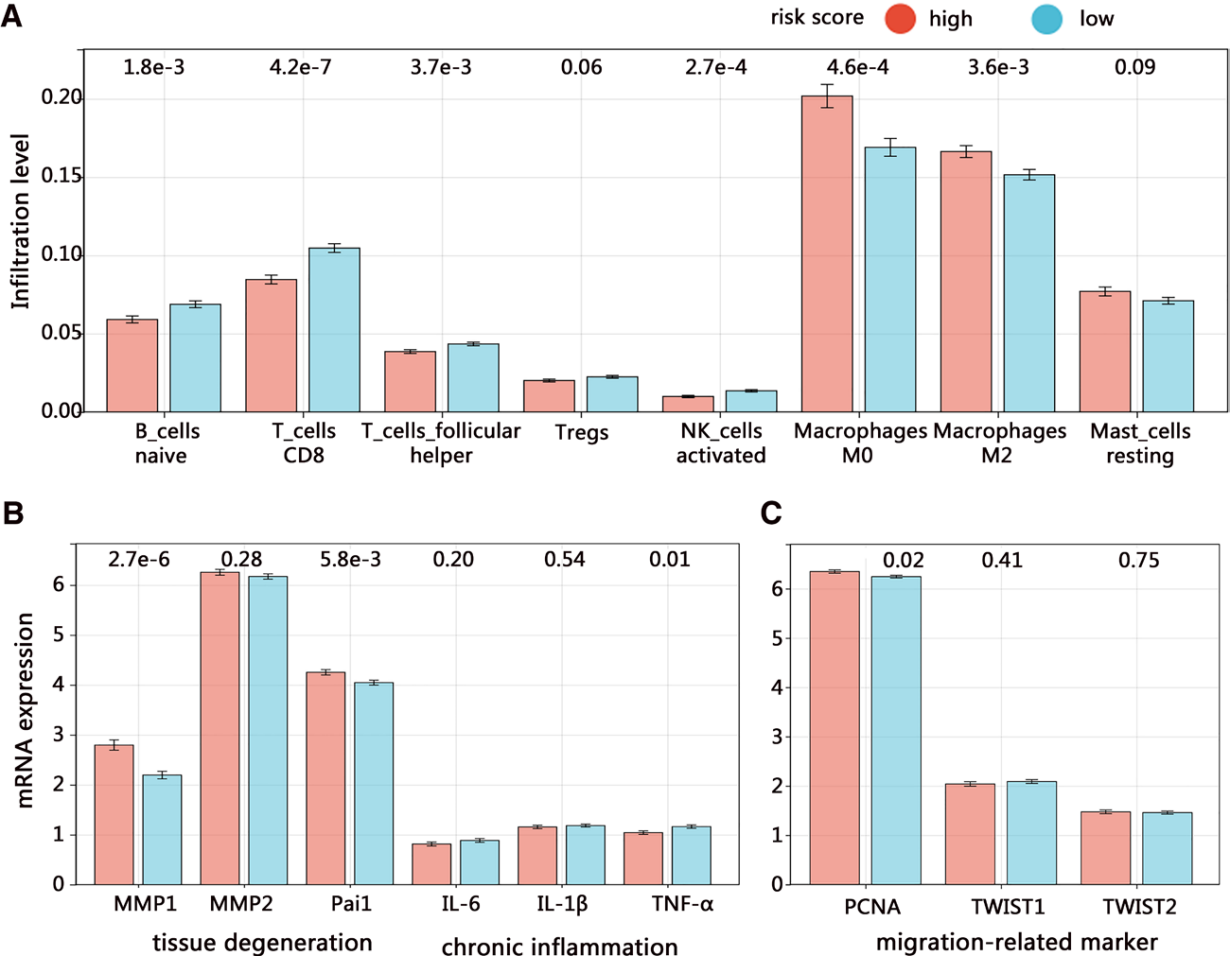
Mechanism exploration of cancer progression associated with cellular senescence. (A) The infiltration level of immune cells. (B) The mRNA expression of tissue degeneration and chronic inflammation-related biomarkers. (C) The mRNA expression of migration and invasion-related biomarkers.

We continuously explored the mechanism of pro-tumorigenesis associated with cellular senescence from aspects of immune cell evasion, tissue degeneration (Pai1, MMP1, MMP2), and chronic inflammation (IL-6, IL-1β, TNF-α). We found that risk score was significantly related to the infiltration level of 8 types of cells among 24 immune cells (data not shown). Further, the infiltration levels of naïve B-cell, CD8 T-cell, follicular helper T-cell, and activated NK-cell were lower in the high-risk score group (Fig. 9A). However, the infiltration levels of M0 and M2 macrophages were higher in high-risk score group. It followed that a high-risk score was related to immune suppression and weak immune surveillance, which may increase the risk of cancer migration.

In addition, we found that the risk score was mainly related to the biomarkers of tissue degeneration rather than the chronic inflammation (Fig. 9B), and the high-risk score group had higher MMP1 and Pai1 expression. These results also implied the risk of cancer migration. Further, we assessed the relationship of risk score with migration-related biomarkers. The result indicated that a higher risk score was related to the higher PCNA expression (migration-related markers), but it did not correlate with TWIST1 and TWIST2 expression (invasion-related markers) (Fig. 9C). These results all indicated the risk of cancer migration associated with senescence.

### 3.6. Clinical validation of 6 signatures in BRCA

**Figure 10.**
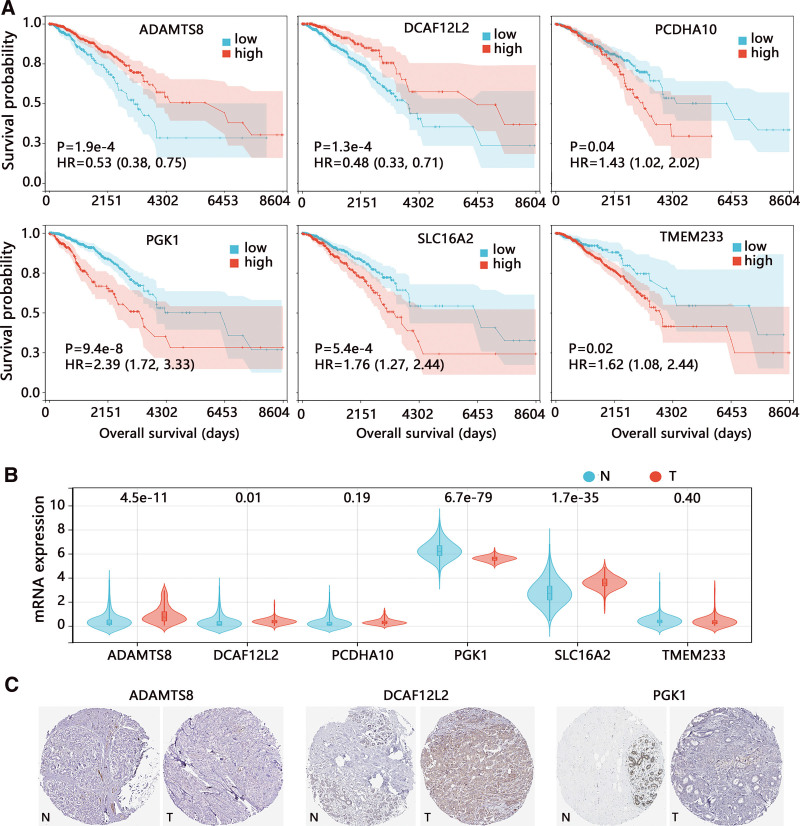
Clinical validation of 6 signatures in BRCA. (A) The correlation of 6 mRNA expression with overall survival time. (B) The mRNA and (C) protein expression difference between normal and tumor tissues. BRCA = breast cancer.

This study finally validated the clinical value of 6 signatures in BRCA. The Kaplan–Meier analysis (Fig. 10A) indicated that patients with higher expression of ADAMTS8 (HR = 0.53) and DCAF12L2 (HR = 0.48) had longer OS time (all *P* < .001). But high expressions of PCDHA10 (HR = 1.43), PGK1 (HR = 2.39), SLC16A2 (HR = 1.76), and TMEM233 (HR = 1.62) were related to the poor prognosis of patients (all *P* < .05). Expression analysis (Fig. 10B) showed that ADAMTS8, DCAF12L2, and SLC16A2 were significantly upregulated, while the PGK1 was downregulated in tumor tissues (all *P* < .05). We found no difference in PCDHA10 and TMEM233 expression between normal and tumor tissues. We also detected the protein expression of 4 differentially expressed genes (Fig. 10C) in tumor and normal tissues, but the SLC16A2 protein was not recorded in HPA.

## 4. Discussion

Cellular senescence has recently been considered a new cancer hallmark, and it plays a complicated role in cancer development due to its divergent effects on tumorigenicity.^[14]^ This study characterized the promising biomarkers regulating cellular senescence in BRCA and explored their influence on BRCA progression. We detected 381 senescence-related genes and identified 41 prognostic biomarkers among them. According to the 41 biomarkers, we clustered the patients into 2 subgroups and then found that 5259 genes were significantly related to the 2 clusters. Through LASSO-Cox regression analyses, a 6-signature risk mode was finally constructed. The validation and comparison analyses all supported the stability and superiority of our risk model for predicting the OS of patients.

Survival analysis showed that a higher risk score correlated with poorer prognosis of patients, and enrichment analysis indicated that the risk score was positively related to glycometabolism such as the TCA cycle in BRCA. The pathway and survival analyses on the risk model all implied the risk of tumor deterioration associated with the senescence-glycometabolism activation axis. Generally, senescent cells showed a slowdown of glucose uptake and metabolism, resulting in less production of ATP.^[15]^ Therefore, inhibition of glycolysis can cause permanent cell cycle arrest by inducing cellular senescence and suppressing cancer development. However, our results suggested the promotion of glycometabolism processes in senescent cells, which contributed to the cancer progression. A previous study also reported the activation TCA cycle in senescent cells. It was found that the activity of pyruvate dehydrogenase kinase (PDK) was simultaneously suppressed in senescent cells.^[16]^ The PDK is the inhibitory enzyme of pyruvate dehydrogenase which plays a vital role in the oxygenolysis of pyruvic acid during the glycolytic pathway. Therefore, the inhibition of PDK increases the activity of pyruvate dehydrogenase, and more glycolytic intermediate of pyruvic acid enters the TCA cycle. Further, the TCA intermediates (citric acid, succinate, ATP) are accumulated. The previous has indicated that the combination of succinate and its receptor (SUCNR1) expressed in the surface of a cancer cell can trigger the PI3K-HIF-1α axis and mediate cancer metastasis by promoting the EMT process.^[17]^ In this part, we clarified the activation of glycometabolism associated with senescence.

**Figure 11.**
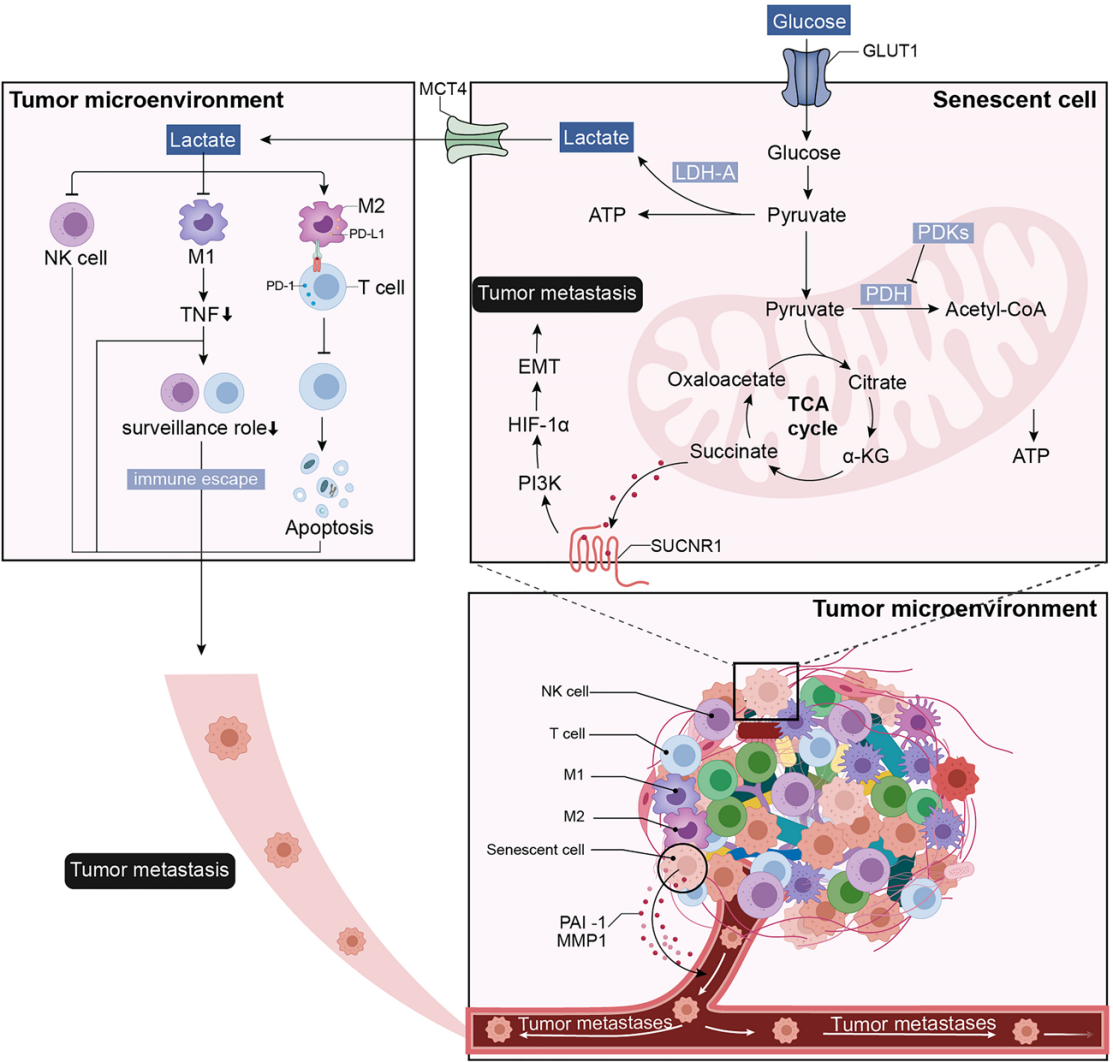
The potential mechanism associated with senescence-related signatures in BRCA. BRCA = breast cancer.

Further, we explored the potential pro-tumorigenesis mechanism associated with the senescence-glycometabolism activation axis. The senescence as a pro-tumorigenic mechanism includes tumor immune evasion, tissue degeneration (Pai1, MMP1, MMP2), and chronic inflammation (IL-6, IB-1β, TNF-α).^[18]^ The correlation of senescence-related signature with immune infiltrates showed that the abundance of CD8 T-cells and activated NK cells decreased, but M2 increased. The cytotoxic CD8 T cells and significant innate lymphoid population such as NK cells all play important antitumor surveillance roles.^[19]^ Our result suggested the deficit immune surveillance caused by the senescence-glycometabolism activation axis. The previous study has reported that glycometabolism products, lactic acid, can cause the immunosuppressive tumor microenvironment by regulating the macrophage polarization to the M2 type^[20]^ and reducing NK cells.^[21]^ The M2 macrophages further expressed the programmed death-ligand 1 (PD-L1) and it combined with PD-1 expressed on the surface of T cells, which promoted the T-cell apoptosis and mediated the immune escape of tumor cells.^[22]^ In addition, M2 macrophages can secrete the IL-10 and induce immunosuppression by suppressing CD8 T-cells.^[23]^ It followed that the accumulation of lactic acid significantly inhibits the tumor surveillance of immune cells^[24]^ and contributed to tumor migration.^[25]^ Our study found a positive correlation of senescence-signature with tissue degeneration proteins of Pai1 and MMP1, which also implied the migration risk of cancer.^[26]^ MMP-1 contributes to the destruction of the extracellular matrix and degradation of the histological barrier of tumor cells, which provides a favorable condition for tumor invasion and metastasis.^[27]^ Although we found no correlation of senescence-related signature with chronic inflammation, we found the decrease of TNF-α expressed by antitumor M1 macrophages, which also supported the risk of immune evasion and tumor migration as TNF is of great importance in CD8 T cell- and NK cell-mediated tumor surveillance.^[28]^ Our study subsequently reported the upregulation of migration-related protein of PCNA. These researches all supported the tumor immune evasion and migration risk associated with senescence-glycometabolism activation axis in BCRA. The potential mechanism was visualized and presented in the following (Fig. 11).

Finally, we clarified the clinical value of 6 signatures in our risk model including ADAMTS8, DCAF12L2, PCDHA10, PGK1, SLC16A2, and TMEM233. ADAMTS8 is one of the ADAMTS family proteins whose structure and function are similar to the matrix metalloproteinases (MMPs).^[29]^ The ADAMTS genes participate in a wide range of biological processes such as extracellular matrix assembly and degradation, and ADAMTS8 has been proven to function as a tumor suppressor gene for BRCA.^[30]^ DCAF12L2 is involved in a variety of cellular processes such as cell cycle progression and apoptosis, and it was only found to correlate with the microsatellite instability in colorectal cancer^[31]^ but no research was reported about BRCA. PCDHA10 has been demonstrated an unusual genomic organization similar to that of B-cell and T-cell receptor gene clusters, and decreased DNA methylation in PCDHA10 has just been found as a risk factor for early-onset high myopia in young children.^[32]^ There was no related research about PCDHA10 in BRCA. PGK1 is an important rate-limiting enzyme in the glycolysis process, and it was regarded as a potential survival biomarker and invasion promoter in BRCA by regulating the HIF-1α-mediated EMT^[33]^ and mediating Warburg effect.^[34]^ Currently, PGK1-related research has been widely reported. For SLC16A2, only the unfavorable prognostic impact in BRCA was revealed.^[35]^ TMEM233 is predicted to be an integral component of the membrane, and it was highly and specifically expressed in SkM, Mb, and Mt.^[36]^ No report about TMEM233 in cancer was observed. It followed that these signatures were significantly related to the risk of tumor migration, conforming to the predicted mechanism associated with senescence in this study.

Finally, several limitations should be noted. In this study, both training set and validation set were obtained from the TCGA. We also explored related datasets containing the prognosis data from the GEO database to verify our risk model. But partial biomarkers were not found in the GEO datasets. Therefore, we did not achieve the external verification of our risk model using GEO data. In addition, this study only focused on the significant biomarker identification associated with cell senescence in BRCA. Finally, 6 genes including the PGK1 and SLC16A2 were determined. However, these genes also play important roles in multiple signaling pathways. Therefore, our study only initially revealed their potential involved in the aspect of cell senescence, and we did not determine whether these genes exert some specific function. Their detailed and specific functions may be verified by the molecular experiment. It must be acknowledged that the lack of experimental validation limits the value of our research to some extent, and related mechanism will be fully verified in our future research.

## 5. Conclusion

Our study established and validated a 6-signature model associated with cellular senescence, and then demonstrated its independent prognostic value in BRCA. The risk model exhibited superior predictive performance in our training, validation, and whole BRCA datasets. Its superiority was also confirmed after comparing it to other published models. This 6-gene signature could be recognized as a molecular biomarker to assess BRCA patients’ prognostic risk. We also revealed the possible senescence-glycometabolism activation axis that acted as a role of pro-tumorigenesis, which may ultimately cause the deficit of immune surveillance and tumor migration. The detailed mechanism needs further verification by molecular biology experiments in future research.

## Author contributions

**Conceptualization:** Xiu-Xia Zhang.

**Data curation:** Xiu-Xia Zhang.

**Investigation:** Xin Yu, Li Zhu, Jun-Hua Luo.

**Methodology:** Li Zhu, Jun-Hua Luo.

**Writing – original draft:** Xiu-Xia Zhang, Xin Yu, Li Zhu.
